# Qilin pills alleviate oligoasthenospermia by inhibiting Bax-caspase-9 apoptosis pathway in the testes of model rats

**DOI:** 10.18632/oncotarget.24985

**Published:** 2018-04-24

**Authors:** Kaishu Zhang, Zhengyan Ge, Longlong Fu, Qi An, Fang Zhou, Ying Guo, Xiaowei Wang, Wenhong Lu, Xiaowei Liang, Shusong Wang, Xuejun Shang, Yiqun Gu

**Affiliations:** ^1^ Chinese Academy of Medical Sciences, Graduate School of Peking Union Medical College, Beijing 100730, China; ^2^ National Health and Family Planning Key Laboratory of Male Reproductive Health, Department of Male Clinical Research, National Research Institute for Family Planning & WHO Collaborating Center for Research in Human Reproduction, Beijing 100081, China; ^3^ Department of Reproductive Medicine, The Affiliated Hospital of Qingdao University, Qingdao 266003, China; ^4^ Institute of Basic Medical Sciences of Xiyuan Hospital, China Academy of Traditional Chinese Medical Sciences, Beijng Municipal Key Laboratory of Pharmacology of Chinese Meteria Medica, Beijing 100091, China; ^5^ Hebei Research Institute for Family Planning, Shijiazhuang 050071, China; ^6^ Department of Andrology, Jinling Hospital Affiliated to Southern Medical University, Nanjing 210002, China

**Keywords:** qilin pills, male infertility, oligoasthenospermia, apoptosis, tripterygium glycosides

## Abstract

At present, the treatment of oligoasthenospermia with western medicine is ineffective. Qilin pill (QLP) is a Chinese traditional medicine for treating male infertility. Recent multicenter clinical studies in China reported that QLPs markedly improved sperm quality. However, the mechanism of action of QLPs on oligoasthenospermia remains unknown. In this study, we investigated the mechanistic basis for improvement of semen parameters and reversal of testis damage by QLPs in a rat model of oligoasthenospermia induced by treatment with tripterygium glycosides (TGs) (40 mg/kg) once daily for 4 weeks. Rats were administered QLPs (1.62 g/kg or 3.24 g/kg) each day for 60 days, with untreated animals serving as controls. The concentration and motility of sperm extracted from rat epididymis were determined, whereas histopathological examination and immunohistochemical apoptosis analysis of rat testes was performed. Expression profiles of apoptosis-related genes were determined by microarray analysis; the results were validated by quantitative real-time PCR, western blotting, and immunohistochemistry. Sperm concentration and motility in the QLP treatment group were increased relative to those in control rats. Testis tissue and DNA damage were reversed by QLP treatment. The improvement function of QLPs on sperm and testis works mainly by suppressing mitochondrial apoptosis in the testis via modulation of B cell lymphoma (Bcl)-2, Bcl-2-associated X protein (Bax), cytochrome C, caspase-9 and caspase-3 expression. QLPs could improve sperm quality and testis damage in a rat model of oligoasthenospermia by inhibiting the Bax-Caspase-9 apoptosis pathway and exerting therapeutic effects.

## INTRODUCTION

Infertility, which is defined by the World Health Organization (WHO) as the inability of a couple to achieve conception or bring pregnancy to term after 1 year or more of regular intercourse without contraception, has become an important reproductive health problem worldwide [1]. It is estimated that male reproductive dysfunction accounts for approximately half of cases of infertility [2], with the main clinical phenotype being idiopathic oligoasthenospermia [3, 4]. The major treatment for idiopathic oligoasthenospermia is still drug therapy. However, the efficacy of western medicine for idiopathic oligoasthenospermia is limited, such that patients with male infertility seek alternative treatment strategies. In Asia, and especially in China, traditional Chinese medicine (TCM) offers alternative and often more effective treatment options, especially with the modernization and increased distribution of TCM compounds [5, 6].

QLPs have been used in TCM for the treatment of oligoasthenospermia and male infertility. Two recent multicenter clinical studies in China reported that QLPs increased sperm concentration and vitality obviously [7, 8]. However, the mechanistic basis for these effects is not well understood. We speculated that it is linked to the regulation of apoptosis during spermatogenesis, and to test this hypothesis, in the present study we examined the effects of QLPs on testis tissue damage and expression of apoptosis-related genes in a rat model of oligoasthenospermia induced by tripterygium glycosides (TGs). We explored the effect of these specific apoptosis pathways on spermatogenesis in the testes of oligoasthenospermia model rats.

## RESULTS

### QLP treatment improves sperm quality and testicular index

There were no significant differences in body weight among the four groups of rats by the end of the experiment. TGs administration reduced testis size relative to control animals, resulting in a decrease in the testicular organ index. In particular, the index of group H (high dose QLPs) was significantly higher than that of group M (oligoasthenospermia with no treatment). Importantly, sperm concentration and motility were lower in group M than in group C (control animals), indicating that the oligoasthenospermia model was successfully established. Sperm concentration and motility were increased by low- and high-dose QLPs administration relative to group M. Sperm concentration but not sperm motility was higher in group H than in group L (low dose QLPs) (*P* < 0.05; Table 1).

**Table 1:** Effect of QLPs on sperm parameters and testicular organ index

Parameter	Group C^†^ (n=10)	Group M (n=10)	Group L (n=10)	Group H (n=10)	ANOVA Value
Sperm paramter					
Sperm concentration (cauda epididymis; 10^6^/ml)	115.80±14.55	19.40±5.50^*^	43.10±17.93^*∆^	58.80±18.15^*∆▲^	F_3,36_ = 75.379 (P<0.0001)
Sperm motility (cauda epididymis; %)	48.20±10.78	4.20±2.04^*^	13.40±5.36^*∆^	14.70±3.89^*∆^	F_3,36_ = 90.665 (P<0.0001)
Testis mass (g)					
Left testis weight	1.83±0.07	0.98±0.17^*^	1.15±0.31^*^	1.36±0.40 ^*∆^	F_3,36_ = 18.677 (P<0.0001)
Right testis weight	1.82±0.08	0.97±0.15^*^	1.15±0.35^*^	1.36±0.37 ^*∆^	F_3,36_ = 18.917 (P<0.0001)
Total testis weight (L+R)	3.65±0.14	1.95±0.30^*^	2.30±0.66^*^	2.72±0.76 ^*∆^	F_3,36_ = 19.244 (P<0.0001)
Body weight (g)	615.40±45.55	616.20±43.65	575.40±59.36	577.80±46.17	F_3,36_ = 2.131 (P<0.113)
Testicular organ index	0.00597±0.000551	0.00320±0.000611^*^	0.00404±0.00130^*^	0.00472±0.00129^*∆^	F_3,36_ = 13.597 (P<0.0001)

^*^P<0.05 vs. group C; ^**∆**^*P*<0.05 vs. group M; ^▲^*P*<0.05 *vs* group L. ^†^Group C, control; group M, oligoasthenospermia; group L, oligoasthenospermia + low-dose QLPs; group H, oligoasthenospermia + high-dose QLPs.

### QLP treatment reverses histopathological damage

**Figure 1:**
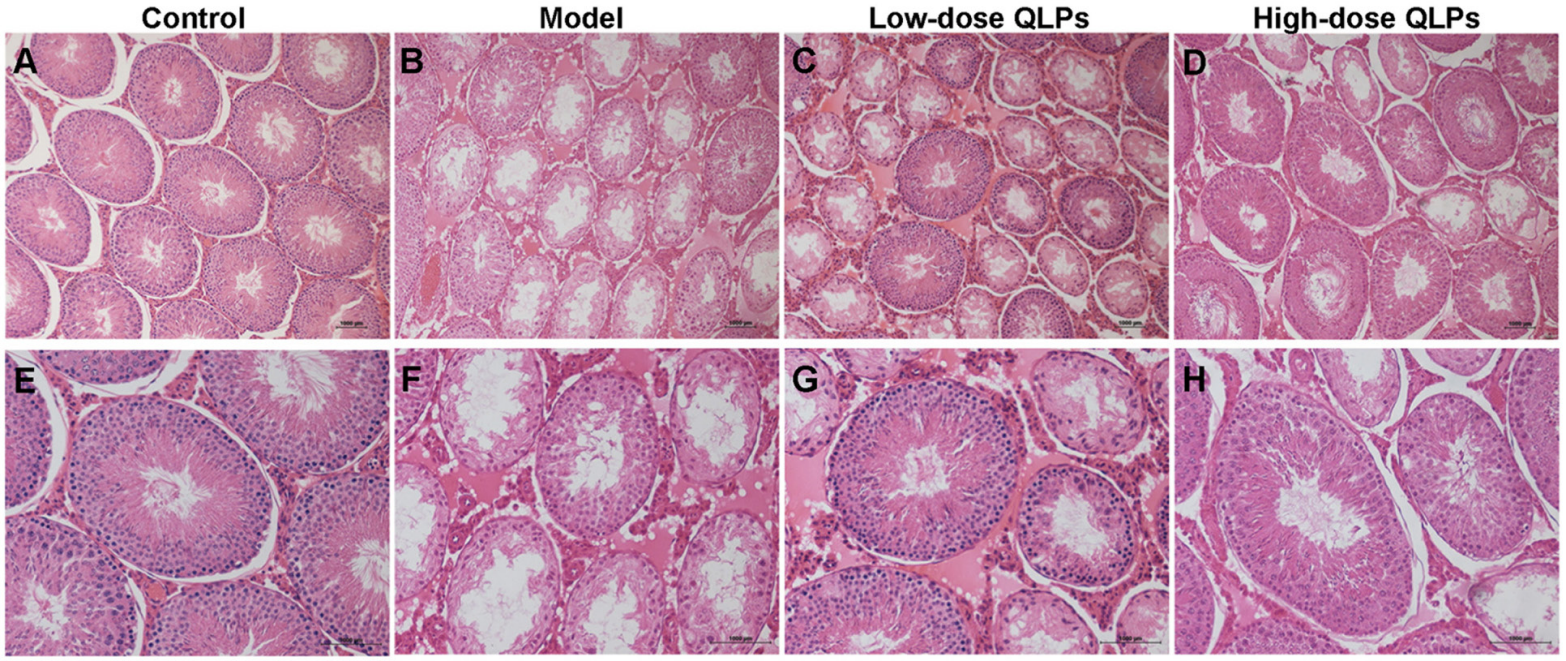
Histological changes in the rat testes Transverse sections of testis tissue were stained with hematoxylin and eosin (HE). Scale bar = 1000 μm. **(A-D)** Magnification: ×100; **(E-H)** Magnification: ×200. Control group with normal histology of seminiferous tubules and interstitium (A&E). Oligoasthenospermia model group with loss of spermatogenic cells, degeneration of interstitial cells, and vacuolization in the cytoplasm of Sertoli cells (B&F). Low-dose QLPs group with increased numbers of spermatogenic cells, hyperplasia of interstitial cells, and decreased number of vacuoles in Sertoli cells (C&G). High-dose QLPs group with further enhancement of tissue recovery as compared to the low-dose QLPs group (D&H).

A histological analysis of testicular tissue from group C revealed a normal process of spermatogenesis, with a regular arrangement of spermatogenic epithelial cells in the seminiferous tubules (Figure 1A & 1E). In contrast, group M exhibited testicular damage including loss, disorganization, and sloughing of spermatogenic cells, degeneration of interstitial cells, and vacuolization in the cytoplasm of Sertoli cells, which were consistent with oligospermia (Figure 1B & 1F). QLPs administration partly restored the morphology of Leydig, Sertoli, and spermatogenic cells (Figure 1C & 1G), with the most dramatic improvement observed in group H (Figure 1D & 1H).

### QLP treatment reduces DNA damage in testis tissue

**Figure 2:**
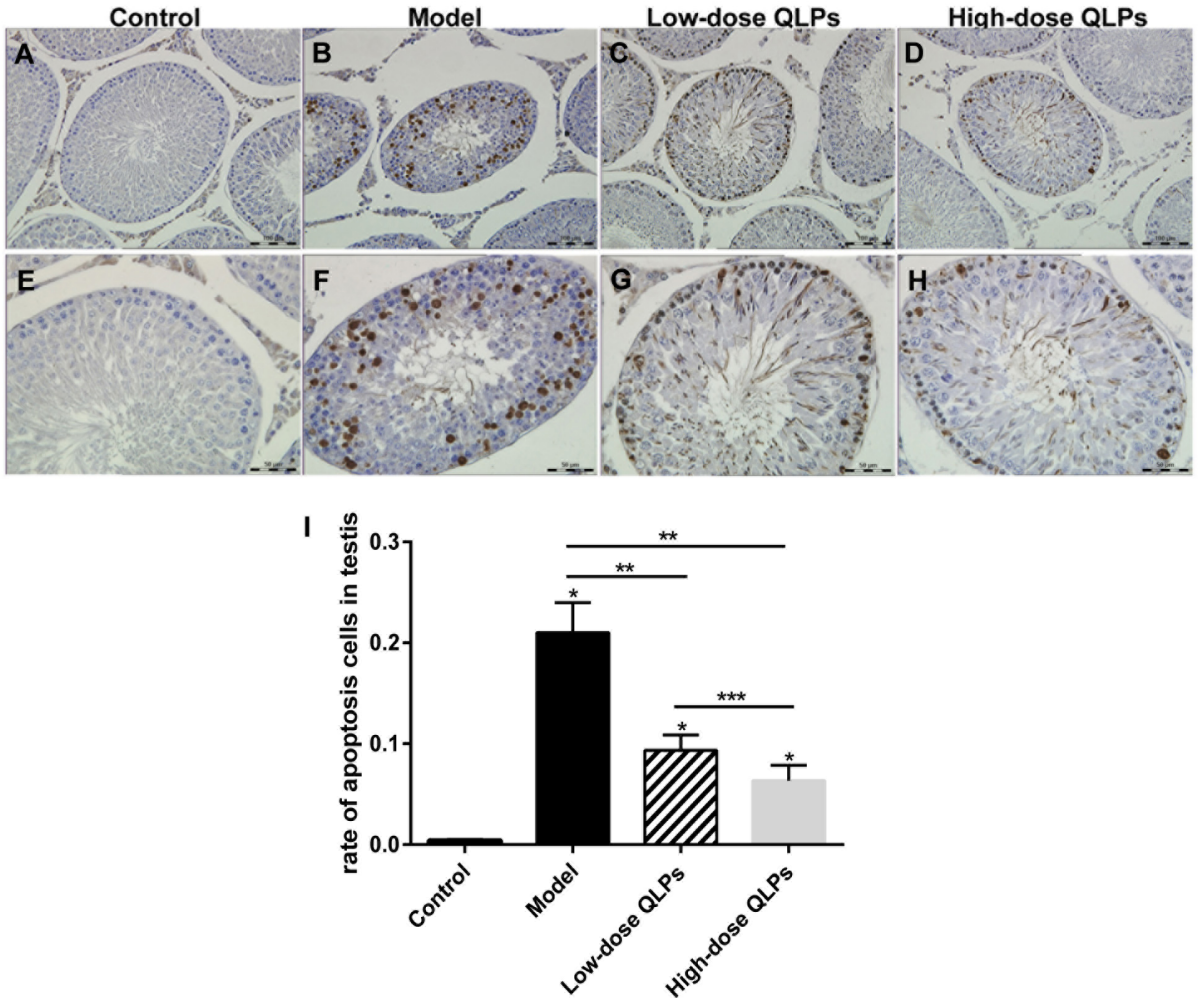
TUNEL staining of apoptotic cells **(A-D)** magnification ×200; **(E-H)** magnification ×400; and **(I)** percentage of apoptotic cells in the seminiferous tubules. All data are presented as the mean ± SEM of at least three experiments. ^*^P<0.05 versus the control group;^*^P<0.05 vs. Control, ^**^P<0.05 vs. Model, ^***^P<0.05 vs. Low-dose QLPs.

Terminal deoxynucleotidyl transferase-mediated dUTP nick end labeling (TUNEL) is an in-situ terminal labeling method for DNA fracture to detect cell apoptosis. In the present study, most of the TUNEL-positive germ cells were detected among the primary spermatocytes and spermatogonia (Figure 2A-2H). Apoptosis in the seminiferous tubules was quantitated using a scoring system based on the percentage of seminiferous tubules with TUNEL-positive cells (Figure 2I) [9]. The percentage of seminiferous tubules with TUNEL-positive cells in groups M, L, and H was higher than that in group C (Figure 2A-2H). There were fewer TUNEL-positive cells in group L and H than in group M.

### QLP treatment alters the expression of apoptosis-related genes

**Figure 3:**
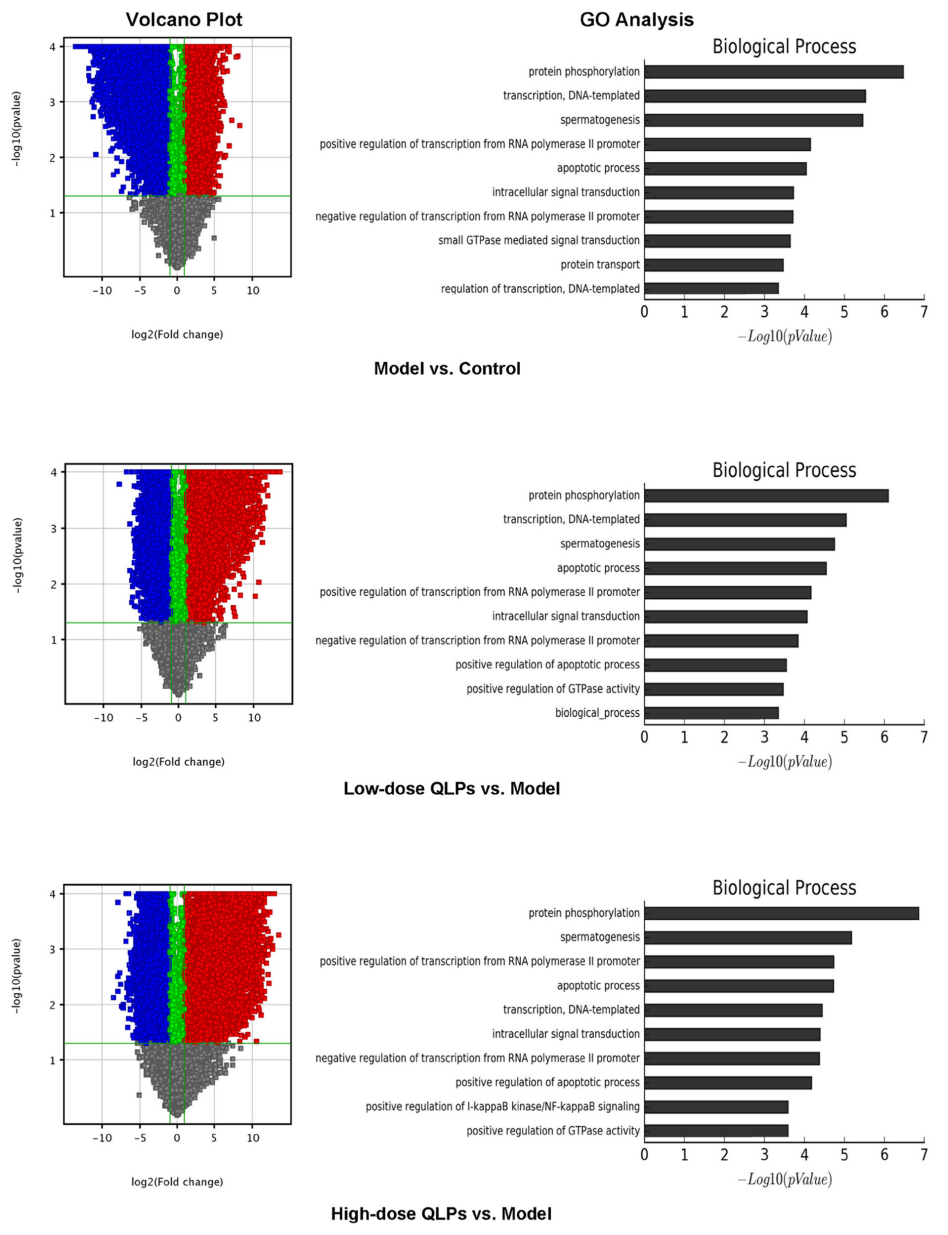
Volcano plot and GO analysis (Left) Volcano plot filtering revealed a greater number of genes that were downregulated (blue) than upregulated (red) in the oligoasthenospermia model as compared to control rats, whereas more genes were upregulated than downregulated in QLP-treated as compared to model rats. (Right) GO analysis revealed the top five biological processes as protein phosphorylation; apoptosis; transcription, DNA-templated; spermatogenesis; and positive regulation of transcription from RNA polymerase II promoter.

We compared gene expression profiles among groups by microarray analysis. As expected, significant differences in gene expression were observed between oligoasthenospermia model rats and controls. In addition, of the 20,912 genes that were analyzed, 8403 and 12,509 were up- and downregulated in group L respectively and 8328 and 11,941 genes were up- and downregulated, respectively, in group H as compared to group M. Only 135 genes were differentially expressed between the two QLPs-treated groups. Volcano plot filtering providing the above difference and GO analysis revealed the top five biological processes associated with differentially expressed genes as protein phosphorylation; apoptosis; transcription, DNA-templated; spermatogenesis, and positive regulation of transcription from RNA polymerase II promoter (Figure 3). We examined apoptosis-related genes that were differentially expressed between group M and groups L and H and found that most were concerned with mitochondrial apoptosis, including Bcl-2, Bax, cytochrome C, and caspase-9, and -3 (Table 2). Studies on the other four biological processes also have been carried out. These results will be published in the relevant articles.

**Table 2:** Gene expression in the groups as determined by gene chip analysis

Contrast groups	Up-regulated	Fold change	Down-regulated	Fold change
Model vs. Control	Bax	8.03	Bcl2111	15.35
	Cycs	4.53	Bcl2112	7.41
	Apaf1	6.61	Bcl2114	92.32
	Casp9	2.16	Bcl2115	6.96
	Casp3	11.53		
Low-dose QLPs vs. Model	Bcl2111	17.94	Bax	7.16
	Bcl2112	6.38	Cycs	3.63
	Bcl2114	88.58	Apaf1	4.29
	Bcl2115	8.26	Casp9	2.13
			Casp3	12.42
High-dose QLPs vs. Model	Bcl2111	19.88	Bax	7.40
	Bcl2112	5.76	Cycs	3.93
	Bcl2114	103.70	Apaf1	6.77
	Bcl2115	9.09	Casp9	3.03
			Casp3	14.12

### Regulation of apoptosis-related genes by QLP treatment

**Figure 4:**
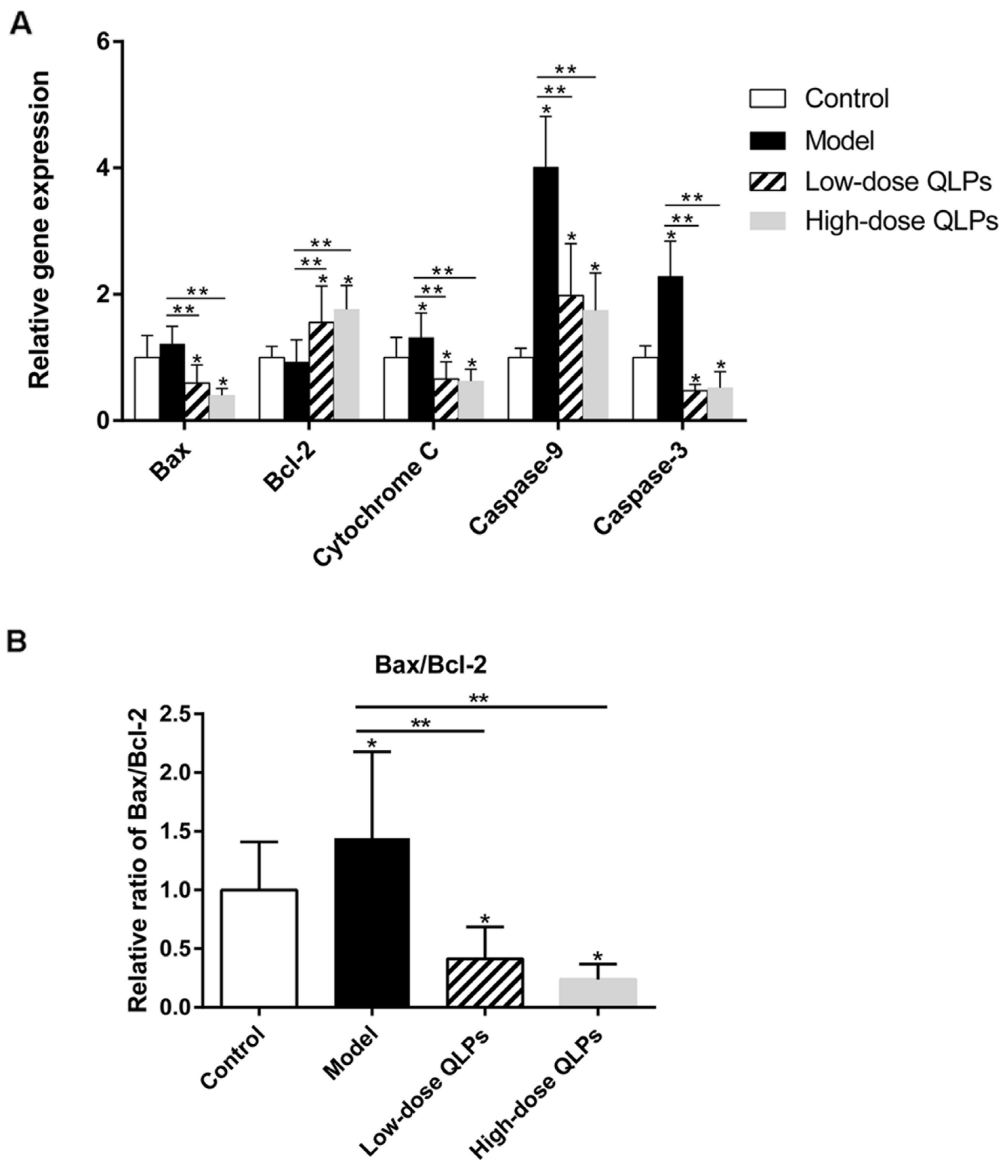
Relative mRNA levels of apoptosis-related genes **(A)** Bax, cytochrome C, and caspase-9 and -3 were upregulated, whereas Bcl-2 was downregulated in oligoasthenospermia model rats; these changes were partly abrogated in rats treated with QLPs. **(B)** Bax/Bcl-2 ratio was increased in the model group and decreased by QLP treatment. Results shown represent the mean ± SEM of three experiments. ^*^P<0.05 vs. Control, ^**^P<0.05 vs. Model, ^***^P<0.05 vs. Low-dose QLPs.

The results of the microarray experiment were validated by qRT-PCR. We confirmed that QLP administration decreased Bax, cytochrome C, and caspase-9 and -3 levels (Figure 4A) as well as the Bax/Bcl-2 ratio (Figure 4B) relative to group M, while increasing the level of Bcl-2 (Figure 4A).

**Figure 5:**
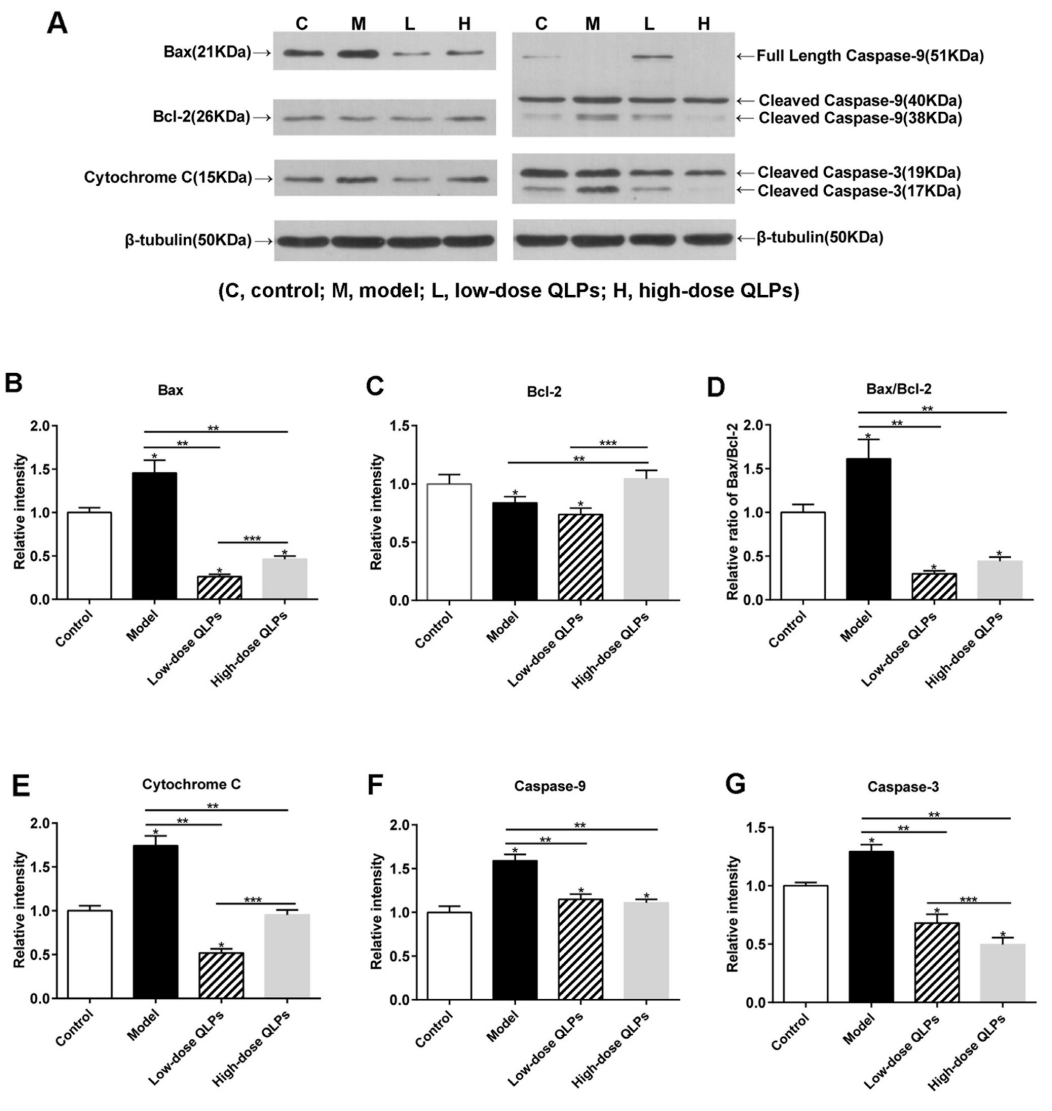
Western blot analysis of apoptosis-related protein expression Results shown represent the average ± SEM from experiments performed in triplicate. **(A)** Equal amounts of protein from testis tissue lysates were analyzed for expression of the indicated proteins. **(B, E-G)** Bax, cytochrome C, and caspase-9 and -3 were upregulated, whereas **(C)** Bcl-2 was downregulated in oligoasthenospermia model rats; this effect was reversed by QLP treatment. **(D)** Bax/Bcl-2 ratio was increased in the model rats and decreased in QLP-treated rats. All data are presented as the mean ± SEM of three experiments. ^*^P<0.05 vs. Control, ^**^P<0.05 vs. Model, ^***^P<0.05 vs. Low-dose QLPs.

A western blot analysis of protein expression showed that Bax, Bcl-2, and cleaved caspase-9 and -3 levels were increased in rats with oligoasthenospermia, and this effect was reversed by QLP treatment (Figure 5A, 5B, 5E–5G). However, in contrast to the results obtained by qRT-PCR, there was no significant difference in the protein level of the anti-apoptotic gene Bcl-2 in the testis between groups M and L (Figure 5C). In addition, the cleaved form of caspase-3 was upregulated in group M (Figure 5G), suggesting activation of the Bax/caspase-9/caspase-3 cascade in oligoasthenospermia.

**Figure 6:**
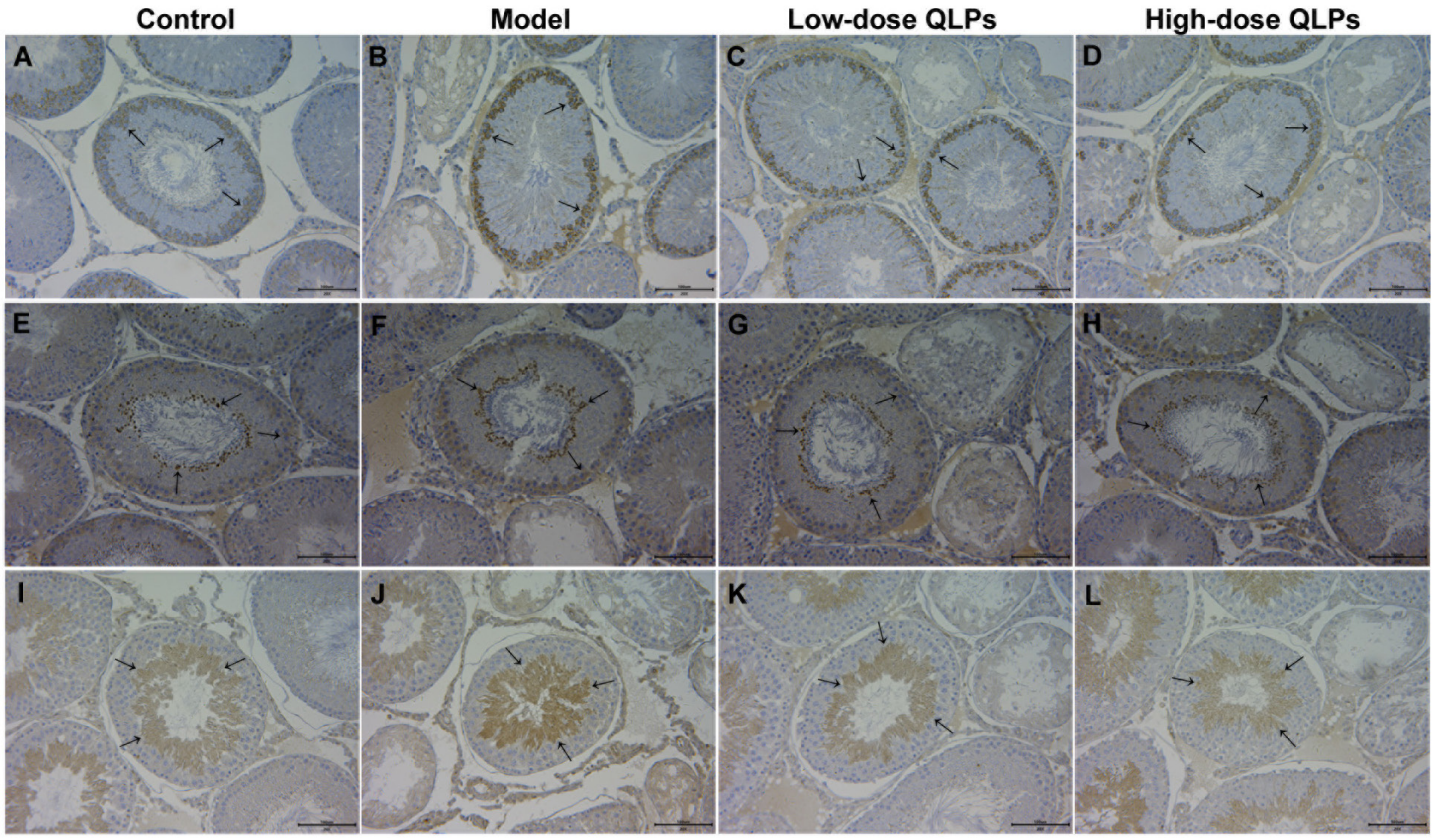
Expression and location of apoptosis-related proteins in testis tissue The expression of Bax **(A-D)**, Bcl-2 **(E-H)**, and cleaved caspase-3 **(I-L)** was detected by immunohistochemistry. Bax and cleaved caspase-3 expression was higher in oligoasthenospermia model rats than in control and QLP-treated groups (B, J). The expression of Bcl-2 was more consistent in control, model, and QLP-treated groups. Bax immunopositivity was detected in spermatogonia and Sertoli cells, Bcl-2 was detected in spermatids and spermatogonia, and cleaved caspase-3 was detected in spermatids, spermatocytes, and Leydig cells.

To identify the cell types undergoing apoptosis in oligoasthenospermia, we examined Bax, Bcl-2, and cleaved caspase-3 expression in testis tissue by immunohistochemistry. Bax was upregulated in spermatogonia and Sertoli cells (Figure 6A–6D); Bcl-2 was expressed in spermatids and spermatogonia (Figure 6E–6H); and cleaved caspase-3 expression was detected in spermatocytes, spermatids, and Leydig cells (Figure 6I–6L). Bax and cleaved caspase-3 immunoreactivity was highest in group M (Figure 6B & 6G) and was decreased in a dose-dependent manner by QLP administration (Figure 6C, 6D, 6K, 6L). The expression difference of Bcl-2 was not obvious in all rats (Figure 6E–6H).

## DISCUSSION

TCM is used for the treatment of oligoasthenospermia in Asia, especially in China [10]. QLP is one of the most effective TCM formulations for male infertility with indications of oligoasthenospermia, sexual dysfunction, or hypogonadism, and has been approved by the China Food and Drug Administration [11–14]. Whether used alone or in combination, QLPs effectively improve semen concentration and activity [7, 8, 13, 14]. However, the exact mechanism by which QLPs affect spermatogenesis has not been fully elucidated. The possible reasons involve spermatogenic cell apoptosis, oxidative stress, and hormonal regulation. The present study examined the effect of QLPs on oligoasthenospermia-induced apoptosis in the testis of model rats.

Many experimental approaches have been used to model spermatogenesis disorders in animals for the study of male infertility, including high temperature, X-ray, or chemicals such as busulfan, cyclophosphamide, ornidazole, and adenine [15–18]. In the present study, we used TGs to establish a rat model of oligoasthenospermia, which presented histopathological changes in the testis, abnormal sperm morphology, and reduced sperm motility observed in human male infertility. In addition, the serum biochemical parameters in the animal model were consistent with those associated with human male infertility [19–21]. TGs induced atrophy of contorted seminiferous tubules and thinning of the seminiferous epithelium, reduced the number of spermatogenic cells, and increased the apoptosis induced by DNA damage in testis tissue, resulting in decreased sperm concentration and motility of sperm in the epididymis. QLPs could reverse the damage to sperm and testis tissue induced by TGs.

Considering that multiple factors (apoptosis, oxidative stress, hormone regulation, and other related causes) regulating spermatogenesis were involved in the therapeutic effect of QLPs on male infertility, microarray was used to screen for the most important apoptosis pathway in treatment of oligoasthenospermia by QLPs. Cell apoptosis associated with spermatogenesis that regulated expression of a series of genes in testis was found to be an important event. The apoptotic genes with great differences in expression were mainly located in the mitochondrial pathway. Therefore, the key genes in this pathway were selected as verification sites, including Bax, Bcl-2, cytochrome C, and caspase-9 and -3.

Cell apoptosis in the oligoasthenospermia model was confirmed by qRT-PCR, western blotting, and immunohistochemistry. We also found that apoptosis-related genes, specifically, those associated with activation of the mitochondrial apoptosis pathway, were upregulated by TG treatment. This effect was partly reversed by QLP administration, which also restored semen quality and testis histology. Mitochondrial apoptosis is controlled by Bcl-2 family proteins, which include Bcl-2 and Bax. The former suppresses apoptosis, whereas the latter antagonizes this cytoprotective effect [22, 23]. These two proteins form hetero- and homodimers in the mitochondrial membrane, and the induction/inhibition of apoptosis depends on the Bax/Bcl-2 ratio [24]. In the present study, we found that Bax and Bcl-2 levels were up- and downregulated, respectively, resulting in an increase in Bax/Bcl-2 ratio in oligoasthenospermia rats. Such an increase has been linked to enhanced mitochondrial membrane permeability and release of cytochrome C into the cytosol, which activates caspase-9 and -3 and leads to apoptosis [25, 26]. Caspase-3 can cleave cellular proteins or activate other caspases via proteolytic cleavage. Caspase-induced DNase activity is associated with DNA degradation and apoptosis [27]. The anti-apoptotic factor Bcl-2 prevents the translocation of Bax to mitochondria, which was observed in our experiments.

The testis contains small convoluted seminiferous tubules and interstitial connective tissue, and is the male sex organ producing sperm and sex hormones and has an important role in maintaining male sexuality. There are mainly three cell types in the testis: spermatogenic cells, Sertoli cells, and Leydig cells. Among them, spermatogenic cells and Sertoli cells constitute the two main cell populations of the seminiferous epithelium in seminiferous tubules. The spermatogenic cells are related to the production of sperm cells. Leydig cells can secrete hormones, and Sertoli cells maintain the structure of the contorted seminiferous tubules, maintain the integrity of the blood-testis-barrier, protect the innate immune privilege in the testis, and control the function of Sertoli cells and Leydig cells. After spermatogenesis, sperm reaches the epididymis via the epididymal duct. Epididymal secretory proteins modify a large number of sperm proteins via phosphorylation, glycosylation, esterification, acylation, or carboxylation, such that sperm gradually acquire various functions and mature [28]. In our study, QLPs affected not only the apoptosis of spermatogenic cells but also the apoptosis of Sertoli cells and Leydig cells. We expect that QLPs improve sperm concentration by inhibiting the apoptosis of spermatogenic cell. Sertoli cells provide physical support and a stable microenvironment for spermatogenesis, promote transport, and provide the necessary cytokines for the development of sperm [29]. Considering the support and protective effects of Sertoli cells on spermatogenic cells, QLPs, which decrease the apoptosis of Sertoli cells, should be able to reduce testicular damage and spermatogenesis disorders caused by oxidative stress or other physical and chemical causes. The inhibitory effect of QLPs on the apoptosis of Leydig cells should affect the secretion of androgen.

Although modulating mitochondrial apoptosis, which is among the most important regulatory mechanisms in spermatogenesis, is one way in which QLPs can alleviate oligoasthenospermia, the contribution of other processes such as oxidative stress, hormonal regulation, and regulation of spermatogenesis-related genes remains to be determined. Relevant experiments are still needed to further verify these unclear mechanisms.

In conclusion, we demonstrated that TGs induce apoptotic cell death in the testes of oligoasthenospermic rats via inhibition of the Bax/caspase mitochondrial apoptosis pathway, and that QLPs exert cytoprotective effects by inhibiting this process. These findings suggest that QLPs are an effective alternative treatment for male infertility.

## MATERIALS AND METHODS

### Chemicals

QLPs were provided by Guangdong Tai’antang Pharmaceutical Co. (Guangdong, China; batch no. B20160605) and comprised 15 herbal ingredients, including prepared fleece flower root, yerbadetajo herb, epimedium herb, dodder seed, Cynomorium songaricum herb, pilose asiabell root, turmeric root tuber, barbary wolfberry fruit, palm leaf raspberry fruit, common yam rhizome, danshen root, milkvetch root, debark peony root, immature tangerine peel, and mulberry fruit [30]. Tablets of TGs were purchased from Shanghai Fudan Fuhua Pharmacy Co. (Shanghai, China; batch no. 160301).

### Animals

Specific-pathogen-free male Sprague-Dawley rats (n = 40; 9 weeks old; 270 ± 10 g) were obtained from Beijing Vital River Laboratory Animal Technology Co. (Beijing, China). Upon arrival, rats were housed under constant environmental conditions (room temperature 23°C ± 2°C on a 12:12-h light/dark cycle at 60% ± 5% humidity) with free access to standard rodent chow and filtered water. The animals were allowed to acclimate for 1 week prior to use in experiments. Experimental procedures were in accordance with the Ethical Principles of Animal Research and were approved by the National Research Institute for Family Planning Ethics Committee for Animal Research.

### Oligoasthenospermia model

The rat model of oligoasthenospermia was established as previously described [31, 32] by oral administration of TGs once daily for 4 weeks at a dose of 40mg/kg/d. Model rats exhibited characteristics of oligoasthenospermia in terms of testicular pathology and semen concentration and motility.

### Experimental groups, treatment, and sample preparation

The rats were randomly divided into four groups of 10 rats each (with use of a computer-generated random number) as follows: control rats (C) were continuously administered physiological saline, and rats in the model (M), low-dose QLP (L), and high-dose QLP (H) groups were first given TGs to induce oligoasthenospermia, followed by physiological saline, 1.62 g/kg QLPs (equivalent to the daily oral dose for patients based on body surface area), and 3.24 g/kg QLPs (double the low dose), respectively, once daily for the subsequent 60 days (equivalent to a cycle of spermatogenesis and maturation of the rats). After the final administration, the rats were weighed and sacrificed, and their testes and epididymides were removed by laparotomy and weighed. The left testis of each animal was fixed in Bouin’s solution and the right testis was flash-frozen in liquid nitrogen and stored at −80°C.

### Analysis of sperm concentration and motility

Sperm concentration and motility were measured with a hemocytometer as previously described [33, 34]. Briefly, the left epididymis of each rat was harvested immediately after sacrifice and cut into small pieces that were transferred to a tube containing 2 mL of warm (37°C) phosphate-buffered saline (PBS) and 1 mL of Medium 199 (Sigma, USA), which was shaken at 37°C for 15 min to allow dispersal of spermatozoa. Approximately 10 μL of diluted sperm suspension was transferred to each counting chamber of the hemocytometer to determine sperm concentration and motility. The latter was measured as the percentage of motile sperm (a + b grade) among total spermatozoa.

### Histopathological analysis

Testis tissue was fixed in Bouin’s solution for 48 h, routinely processed with an automatic tissue processor, and embedded in paraffin. The tissue block was cut into sections at a thickness of 5 μm that were stained with hematoxylin and eosin according to standard procedures and examined under a light microscope (Nikon Eclipse TS100, Japan).

### TUNEL staining

Apoptotic detection (TUNEL analysis) was performed on Bouin’s-fixed 5-μm paraffin sections using the In Situ Cell Death Detection Kit (POD, Roche). Briefly, after dewaxing and rehydration, the tissue sections were incubated with 25 mg/l proteinase K for 20 min at 37°C. Nonspecific labeling was blocked by preincubating the sections with 3% H_2_O_2_ in methanol for 10 min at room temperature. To each section, 50 μl of TUNEL reaction mixture was added, and the sections were incubated for 60 min at 37°C in a humidified atmosphere. After the sections were rinsed, 50 μl of converter-POD was added onto each section, and the secions were incubated for 30 min in a humidified chamber at 37°C. The sections were rinsed again and incubated with 100 μl of DAB substrate for 10 min at room temperature. Color development was stopped by washing the sections with PBS. The sections were counterstained with hematoxylin. For the negative control, the TUNEL reaction mixture was replaced with the same volume of reaction buffer. The percentage of seminiferous tubules containing brown-stained cells was analyzed.

### RNA isolation and microarray analysis

Total RNA was extracted from 12 testis tissue samples (three samples per group). RNA quantity and quality were evaluated with the NanoDrop ND-2000 system (Thermo Fisher Scientific, USA), and RNA integrity was assessed with the Bioanalyzer 2100 system (Agilent Technologies, USA). RNA samples with RNA integrity value > 9.0 (average value: 9.6) were used for analysis. Sample labeling and microarray hybridization were performed according to the manufacturer’s protocols. Briefly, total RNA was transcribed to double-stranded cDNA, which was labeled with cyanine-3-CTP. Labeled cRNAs were hybridized to a SurePrint G3 Rat GE v.2.0 Microarray (8×60K, design ID: 074036) (Agilent Technologies). The array was washed and scanned with the G2505C Scanner (Agilent Technologies). Feature Extraction software v.10.7.1.1 (Agilent Technologies) was used to analyze array images and obtain raw data. Genespring v.13.1 (Agilent Technologies) was used for analysis of raw data, which was performed by OE Bio-tech (Shanghai, China). Genes that were differentially expressed between treatment and control groups were identified by volcano plot filtering according to fold change and P value, which was calculated with t tests. The threshold for up- and downregulated genes was fold change ≥ 2.0 and P ≤ 0.05. Gene ontology (GO) analysis was performed to clarify the significance of differentially expressed mRNAs by the standard enrichment computation method. Differentially expressed apoptosis-related genes identified by microarray analysis were validated by quantitative real-time (qRT-) PCR, western blotting, and immunohistochemistry.

### qRT-PCR

The expression of apoptosis-related genes was evaluated by qRT-PCR. Total RNA was isolated from frozen rat testis tissue using TRIzol reagent (Invitrogen, USA). The quality of extracted RNA was verified by agarose gel electrophoresis and the RNA was used to synthesize cDNA using the PrimeScript RT reagent kit with gDNA Eraser (Takara Bio, Japan). Target gene-specific primers (Table 3) were synthesized by Invitrogen. Glyceraldehyde 3-phosphate dehydrogenase (GAPDH) served as an internal control. PCR amplification was performed on a 7500 real-time PCR system (Applied Biosystems, USA). The 20-μl reaction mixture contained 10 μl of SYBR Premix Ex Taq II (Tli RNaseH Plus) ROX Plus (Takara Bio), 10 μl of cDNA (1:5 dilution), and each primer at 0.5 mmol/l. The reaction conditions were as follows: 95°C for 10 min, followed by 40 cycles of 95°C for 5 s and 60°C for 40 s. Melting curves were generated with a three-segment cycle of 95°C for 10 s, 60°C for 60 s, and 95°C for 15 s in the continuous acquisition mode. PCR reactions were performed in triplicate. The cycle counts for PCR products were normalized to those for *GAPDH*. A negative control consisting of water was included in each reaction set. To determine PCR efficiency, a standard curve was generated for each run by plotting the crossover point against the log (number of starting molecules) of serially diluted cDNA. The specificity of each primer was assessed by melting curve analysis. Data were analyzed with the 2^−ΔΔCt^ method.

**Table 3:** Primer-specific conditions used for PCR amplification of candidate genes

Gene	Primer sequence	Amplicon size	Tm
Bax	F: 5’- GGTGGTTGCCCTTTTCTACTTTGC -3’	113	60
	R: 5’- GCTCCCGGAGGAAGTCCAGTG -3’		
Bcl-2	F: 5’- GGGCTACGAGTGGGATACTGGAG -3’	101	60
	R: 5’- CGGGCGTTCGGTTGCTCT -3’		
Cytochrome C	F: 5’-TCGAGGTCATGGAGAGAAATGA-3’	227	60
	R: 5’-AGTTGCAAGGACGTGCAGTTTA-3’		
Caspase-9	F: 5’- GAGCCAGATGCTGTCCCATACCA -3’	110	60
	R: 5’- GGGAAGGTGGAGTAGGACACAAGG -3’		
Caspase-3	F: 5’- ACTGGAAAGCCGAAACTCTTCATCA -3’	127	60
	R: 5’- GGAAGTCGGCCTCCACTGGTATC -3’		
GAPDH	F: 5’-TTCCTACCCCCAATGTATCCG-3’	270	60
	R: 5’-CCACCCTGTTGCTGTAGCCATA-3’		

### Western blotting

Frozen testis tissue was homogenized in ice-cold radioimmunoprecipitation assay buffer (pH 7.5) containing protease inhibitor cocktail (Roche, USA). After denaturation at 95°C for 5 min, samples were centrifuged at 12,000 × *g* for 20 min at 4°C and the supernatant was collected and stored at −20°C. Proteins were separated by 10% sodium dodecyl sulfate–polyacrylamide gel electrophoresis and transferred to a polyvinylidene difluoride membrane that was blocked overnight at 4°C in PBS containing 0.1% Tween 20 (PBS-T) and 5% skim milk, and then probed with rabbit anti-rat antibodies against the following proteins: Bcl-2 (1:1000; ab59348) and Bax (1:1000; ab32503) (both from Abcam, USA); cytochrome C (1:500; sc13156) (Santa Cruz Biotechnology, USA); cleaved caspase-9 (1:1000: #9506) and cleaved caspase-3 (1:500; #9664) (both from Cell Signaling Technology, USA); and β-tubulin (1:5000; YM3030) (Immunoway, USA). After three washes with TBS-T, the membrane was incubated at room temperature for 1 h with horseradish peroxidase (HRP)-conjugated goat anti-rabbit secondary antibody (1:1000; Cell Signaling Technology) in PBS-T with 2% skim milk and washed three times with TBS-T [35, 36]. Immunoreactivity was visualized by enhanced chemiluminescence. The relative signal intensity of protein bands was analyzed with Image J software (National Institutes of Health, USA).

### Immunohistochemistry

Rat testis tissue was fixed in Bouin’s solution and embedded in paraffin, and cut into sections with a thickness of 5 μm that were collected on glass slides. Sections were deparaffinized in xylene, rehydrated through graded series of ethanol, and rinsed with water [37]. Endogenous peroxidase activity was blocked by incubation in 0.3% hydrogen peroxide in PBS for 30 min at room temperature. Slides were blocked for 1 h in PBS supplemented with 10% normal goat serum. Bcl-2, Bax, and cleaved caspase-3 expression was detected by overnight incubation at 4°C with antibodies against Bax (1:100), Bcl-2 (1:50), or cleaved caspase-3 (1:100). After washing, sections were incubated for 1 h with HRP-conjugated goat anti-rabbit IgG (1:2000; Santa Cruz Biotechnology, USA) in 10% goat serum, then counterstained for 10 s with hematoxylin (Gill no. 3; Sigma, USA) and examined with a light microscope (Nikon Eclipse TS100, Japan).

### Statistical analysis

Data are expressed as the mean ± SD and were analyzed with SPSS software (version 22.0; SPSS, Chicago, IL, USA). Differences between group means were assessed by one-way analysis of variance (ANOVA) followed by the Student-Newman-Keuls test. *P* < 0.05 was considered significant.
